# Impact of socioeconomic status on patient experience on quality of care for ambulatory healthcare services in tertiary hospitals in Southeast Nigeria

**DOI:** 10.1186/s12913-020-05332-0

**Published:** 2020-05-26

**Authors:** Henry E. Aloh, Obinna E. Onwujekwe, Obianuju G. Aloh, Ijeoma L. Okoronkwo, Chijioke Joel Nweke

**Affiliations:** 1 Health Economics and Policy Research Unit, Department of Health Services, Alex Ekwueme Federal University Ndufu-Alike Ikwo, Ikwo, Ebonyi State Nigeria; 2 Department of Health Administration & Management, Faculty of Health Sciences, College of Medicine, University of Nigeria, Enugu Campus, Enugu, Nigeria; 3 Primary Health Development Agency, Ministry of Health, Enugu, Ebonyi State Nigeria; 4 Department of Mathematics/Computer Sciences/Statistics & Informatics, Alex Ekwueme Federal University Ndufu-Alike Ikwo, Enugu, Nigeria

**Keywords:** Quality of care, Patient experience, Socioeconomic status, Hospitals, Nigeria

## Abstract

**Background:**

To determine how socioeconomic factors, such as level of education and employment status, affect patient experiences on quality of care for ambulatory healthcare services in teaching hospitals in southeast Nigeria.

**Methods:**

The study is of a cross-sectional design and exit poll was used to collect its data. A pre-tested structured questionnaire was administered to clients accessing care in the outpatient departments of three tertiary hospitals in Nigeria. The assessment of patient experiences for quality of care was based on five (5) domains of care: waiting time; environment of the outpatient department; quality of doctor’s care; quality of care by nurses/other health workers; and responsiveness of care. In addition, the overall quality of care was assessed.

**Results:**

The mean rating of patient experience for quality of care for ambulatory healthcare services (outpatients’ care) was 74.31 ± 0.32%. Moderate differences were observed between the hospitals assessed for various levels of patients’ care, especially for waiting time, quality of doctors’ care and overall quality of care. Employment status was a statistically significant (*p* ≤ 0.05) determinant of overall patient experience rating for quality of care, while the level of patient’s education was an influence on the perception of waiting by the patients and their rating of care from nurses/other healthcare providers (apart from medical doctors).

**Conclusion:**

The study showed that educational and employment status (measures of socioeconomic status) of patients determined how patients receiving ambulatory (outpatient) healthcare services perceived the quality of care in the hospitals. Hence, in order to ensure equity, there is need to institutionalize patient-centered care, while full consideration is given to the patients’ socioeconomic status.

## Background

People’s state of health and the manner in which it is cared for is indeed regarded as a very important component of economic development of any country [1, 2]. The health sector targets often relate to traditional hospital functions such as diagnosis, outpatient treatment and inpatient care. Outpatient services are those hospital services that do not require an overnight stay and may include care such as diagnostic tests, prescriptions or simple treatment/procedures [3]. These services are offered on ambulatory basis, and they account for the majority of patient-medical professional encounters than any other hospital services (approximately four times than that of inpatients).

Few studies have examined patient experience for quality of care in resource-scarce environment like sub-Saharan African countries such as Nigeria. Measurement of quality can be very useful to stakeholders who choose between different health care providers, and to the policy makers who strive to enunciate better health policies [4]. Standardized surveys of patients and relatives can reliably measure hospital performance by assessing patients’ experience and outcomes of treatment. Patient experience (PE) evaluates what happens at the point of contact between the patient, the practice, and the provider [5]. It captures health system responsiveness, including the manner and environment in which people are treated when they seek healthcare [6]. It also includes sufficient information and education of the patient, coordination of care, physical comfort, emotional support, respect for patient preferences, involvement of family and friends [4].

Patient experience is not the same as perceived quality which is predominantly a cognitive assessment of what happened and how it happened [7]. It is also different from patient satisfaction which is referred to as patients’ emotion, feelings and their perception of delivered healthcare service [8]. Patient satisfaction is regarded as the degree of congruency between patient expectations of ideal care and their perception of the real care received [9] and it tends to seek subjective responses from patients. In contrast, PE asks patients to give factual responses to questions about what did or did not happen during episode of care.

A major challenge to measuring healthcare quality in low- and middle-income countries (LMICs) was highlighted by a study carried out by Dunsch et al., (2017) on more than 2200 patients in Nigeria using positive and negative framed satisfaction statements [10]. The result showed that patient satisfaction measurements are deeply sensitive to the framing of the questions and hence the need to adapt patient experience that avoids a short-laced response of agree/disagree and yes/no questions. It is believed that PE survey questionnaires that are well designed and appropriately administered can provide a robust measure of quality of care and reliably measure hospital performance against explicit standardization [11, 12]. Several studies have been carried out on quality of healthcare services especially in developed countries; quite a handful in African region and very few in sub-Saharan African countries like Nigeria. Many reviewed literatures on quality of healthcare show that waiting time is one of the most important indicators or variables of healthcare quality. One of such is a cross sectional observational study by the Patels (2017) on waiting time and outpatient satisfaction at Gujarat medical education research society hospital India, using 135patients from the outpatient department (OPD) [13]. A cross sectional survey of nurses (33,659) and patients (11,318) in 12 countries in Europe and the United States on patient safety, satisfaction and quality of hospital care, using hierarchical modelling and robust logistic regression, showed that deficits in hospital care quality were common in all countries [14]. Mejabi et al., (2008) in their work on dimensions of hospital service quality in Nigeria, as published in European Journal of Social Sciences, used probability and quota sampling methods on 6 service points (medical outpatient clinic, surgical outpatient clinic, medical male ward, medical female ward, surgery male ward and surgery female ward) and applied correlation matrices/factor analysis to evaluate quality of healthcare in two Nigeria teaching hospitals. Their findings show that eight (8) dimensions of resource availability, quality of care, condition of the clinic/ward, condition of the facility, quality of food, attitude of doctors and nurses, attitude of non-medical staff and waiting time adequately describe service quality phenomenon in Nigerian hospital setting [15].

The aim of the present study is to evaluate patients’ experience (PE) on quality of care among clients accessing ambulatory (outpatients) healthcare services in teaching hospitals, Southeast Nigeria.

There is paucity of knowledge on the effects of socio-economic variables on PE for ambulatory healthcare services in hospitals. The present study focused on how socio-economic factors represented, in the context of this study, by level of education and employment status of outpatients affect PE on quality of care. Level of education and employment status were used because the two are the commonest socio-economic parameters that are used by the teaching hospitals in Nigeria when disaggregating socio-economic status of their patients.

## Methods

The study design was a cross-sectional exit survey. Three (3) tertiary hospitals were randomly selected from seven (7) teaching hospitals in Southeast, Nigeria. The study population was 7847 patients, which was the projected outpatients flow for the 3 hospitals over a period of 3 months the data collection lasted. The hospitals are: Enugu State University Teaching Hospital (ESUTH), University of Nigeria Teaching Hospital (UNTH) and Federal Teaching Hospital Abakaliki (FETHA).

The sample size was determined using a formula for calculating required sample size for a patient population of less than 10,000 persons. N_f_ = n/1 + (n/N); N is average number of target population. Value of n was calculated using: *n* = Z^2^pq/d^2^ as the formula [16]. Where n is minimum sample size required, Z is normal standard deviation at 95% confidence level and is 1.96, p is prevalence of the factor under study (in this case the outpatients) and from previous studies it is 84% (0.84) for outpatients [17].

A pre-tested structured patient experience questionnaire (PEQ) with scores assigned to the questions was developed and used for this study. The PEQ is designed to elicit actual experiences, ranging from the duration of hospital’s waiting time to the environmental condition of the outpatient department, the quality of care rendered by the doctors and other health workers, the responsiveness of care and indeed the patient’s overall rating of his experience [18]. The study generated and attached relative weights to each question as an indirect measure of the quality of ambulatory healthcare services. This was based on plausible measurements from previous studies that were adopted for the context of the study area [18–20]. The weights in the present study were represented by scores, ranging from zero to ten (0–10).

The questionnaire was pre-tested in a secondary level hospital that is located within the same geographical region, using about 10% of the sample size. The instrument was then revised for language and better understanding. Hence, there was no obvious problem with the respondents comprehending the questions and providing plausible answers to the questions. A reliability test for the questionnaire was carried out for internal and external consistency using Cronbach’s alpha test and this gave Cronbach’s alpha coefficient of 0.632 (i.e. > 0.5), showing that the questionnaire was consistent and therefore reliable. A total of 422 questionnaires were administered to respondents (outpatients) in the teaching hospitals by well-trained interviewers. The responses were elicited without patient’s identifier after a written consent were obtained from respondents [18, 20].

The data were analyzed using statistical methods such as Z-test and ANOVA to examine mean differences, and Chi-square/Log Linear association tools to compare the relationship between PE variables with level of education and employment status.

## Results

Out of the 422 questionnaires that were administered a total of 408 were fully responded to. This represents a response rate of 96.7%. The study results show that 50.7% of the respondents were male, whilst 49.3% were female. Majority (73%) of the patients were within active age range of 18–65 years. About 24% were above 65 years and 2.9% were below 18 years of age. It was found that 40.7% of the respondents had university education and 25.2% had high school education, whilst the remaining 34.1% had either primary school education or no formal education. Less than half (about 41.7%) were employed, while 28.2% were unemployed and 18.1% retirees that were no longer economically active. Table 1 also shows that slight majority (51%) of the patients received healthcare services at the surgical outpatient department (SOPD), while 49% were cared for in medical outpatient department (MOPD).

**Table 1:** Demography and socioeconomic status of outpatients

Variable	Categories	Hospital			
		FETHA	ESUTH	UNTH	Total
Sex	Male	87(55.1)	67(49.3)	53(46.5)	207(50.7)
	Female	71(44.9)	69(50.7)	61(53.5)	201(49.3)
Marital Status	Married	110(69.6)	98(72.1)	71(62.3)	279(68.4)
	Not Married	41(25.9)	32(23.5)	40(35.1)	113(27.7)
	Others	7(4.4)	6(4.4)	3(2.6)	16(3.9)
Age (Years)	<=18	6(3.8)	2(1.5)	4(3.5)	12(2.9)
	19–64	112(70.9)	96(70.6)	90(78.9)	298(73)
	65+	40(25.3)	38(27.9)	20(17.5)	98(24)
**SES**					
Level of Education	No Formal Education	23(14.6)	15(11)	16(14)	54(13.2)
	Primary Education	36(22.8)	26(19.1)	23(20.2)	85(20.8)
	Secondary Education	50(31.6)	29(21.3)	24(21.1)	103(25.2)
	University Education	49(31)	66(48.5)	51(44.7)	166(40.7)
Employment Status	Employed	68(43)	56(41.2)	46(40.4)	170(41.7)
	Not Employed	37(23.4)	34(25)	44(38.6)	115(28.2)
	Student	22(13.9)	13(9.6)	14(12.3)	49(12)
	Retired	31(19.6)	33(24.3)	10(8.8)	74(18.1)
	**Total**	**158(100)**	**136(100)**	**114(100)**	**408(100)**

The figures in the bracket, (), are % along the column

Table 2 shows that there were significant statistical differences (*p* ≤ 0.05) among hospitals for mean patient experience (PE) scores on waiting time, quality of doctors’ care and that of overall rating. FETHA had the highest mean PE score for quality of doctor’s care (43.89 ± 7.04) and for overall PE rating (76.46 ± 11.08%), UNTH had the best score for waiting time PE score (11.32 ± 5.73).

**Table 2:** Analysis of mean difference for patient’s experience variables

Variable	Hospital				
	FETHA	ESUTH	UNTH	***p***-value	Remark
Waiting Time	10.56^b^ ± 4.96	7.67^a^ ± 4.23	11.32^b^ ± 5.73	0.000	*Significant*
OPD Environment	26.21^a^ ± 4.42	26.64^a^ ± 4.95	27.09^a^ ± 4.10	0.285	*Not Significant*
Quality of Doctors Services	43.89^c^ ± 7.04	41.63^b^ ± 9.23	38.66^a^ ± 9.74	0.000	*Significant*
Quality of care by Nurses/Other Medical Staff	22.04^a^ ± 7.23	19.89^a^ ± 6.60	19.95^a^ ± 7.67	0.172	*Not Significant*
Responsiveness	48.83^a^ ± 13.38	51.07^a^ ± 10.86	48.82^a^ ± 15.93	0.282	*Not Significant*
Overall Quality Rating (%)	76.46^b^ ± 11.08	72.90^a^ ± 11.89	73.00^a^ ± 12.39	0.014	*Significant*

Values are mean ± Standard Deviation. Mean values along the row with different alphabetical superscript indicates a significant different

Table 3 disaggregates the healthcare services into medical and surgical, and compared the differences in their mean PE scores, within and between the hospitals. FETHA’s MOPD had mean PE score of 27.22 ± 0.43 points for its environment, better than that of its SOPD which had mean PE score of 25.36 ± 0.52 points. FETHA’s MOPD overall PE rating was 78.37 ± 1.14% and significantly higher than that of SOPD (74.85 ± 1.29). Similarly, in ESUTH the MOPD services were significantly (*p* ≤ 0.05) better than those of SOPD for quality of doctor’s care, quality of care by nurses/other health professionals, as well as for overall rating. Only UNTH’s SOPD overall PE rating of 76.96 ± 1.51% was significantly (p ≤ 0.05) better than that of its MOPD (68.43 ± 1.58%).

**Table 3:** Analysis of mean difference for patient’s experience variables scores among outpatients, along type of care (surgical and medical)

Variable	Service Type	Hospital			***P***-value	Remark	Overall
		FETHA	ESUTH	UNTH			
Waiting Time	Medical	11.0^b^ ± 0.53	7.4^a^ ± 0.47	11.67^a^ ± 0.91	*0.000*	*Significant*	9.75^a^ ± 0.38
	Surgical	10.18^b^ ± 0.58	8.0^a^ ± 0.56	11.31^b^ ± 0.62	*0.001*	*Significant*	9.88^a^ ± 0.35
	*p*-value	0.290	0.417	0.749			0.801
OPD Environment	Medical	27.22^a^ ± 0.43	26.44^a^ ± 0.52	25.85^a^ ± 0.55	*0.170*	*Insignificant*	26.57^a^ ± 0.29
	Surgical	25.36^a^ ± 0.52	26.89^b^ ± 0.70	28.16 ^b^ ± 0.498	*0.002*	*Significant*	26.63^a^ ± 0.34
	*p*-value	0.007*	0.611	0.002*			0.885
Quality of Doctors’ care or Services	Medical	44.63^b^ ± 0.68	43.75^b^ ± 0.86	35.25^a^ ± 1.60	*0.000*	*Significant*	41.8^a^ ± 0.57
	Surgical	43.27^a^ ± 0.85	39.03^a^ ± 1.34	41.62^a^ ± 1.90	*0.028*	*Significant*	41.54^a^ ± 0.66
	*p*-value	0.216	0.004*	0.026*			0.761
Quality of care or Services by Other Medical Staff	Medical	22.71^a^ ± 1.17	22.4^a^ ± 1.13	19.23^a^ ± 1.63	*0.142*	*Insignificant*	21.62^a^ ± 0.78
	Surgical	21.64^b^ ± 1.01	16.47^a^ ± 1.81	20.88^b^ ± 1.90	*0.065*	*Insignificant*	20.60^a^ ± 0.82
	*p*-value	0.49	0.010*	0.513			0.371
Responsiveness	Medical	50.00^b^ ± 1.29	50.53^b^ ± 1.40	42.92^a^ ± 2.38	*0.003*	*Significant*	48.33^a^ ± 0.97
	Surgical	47.85^a^ ± 1.63	51.72^b^ ± 1.17	53.93^b^ ± 1.62	*0.016*	*Significant*	50.77^a^ ± 0.91
	*p*-value	0.302	0.515	0.000*			0.066
Overall Rating (in %)	Medical	78.37^c^ ± 1.14	74.53^b^ ± 1.22	68.43^a^ ± 1.58	*0.000*	*Significant*	74.30^a^ ± 0.79
	Surgical	74.85^b^ ± 1.29	70.89^a^ ± 1.68	76.96^b^ ± 1.51	*0.020*	*Significant*	74.31^a^ ± 0.86
	*p*-value	0.043*	0.041*	0.000*			0.993

Values are mean ± Standard Deviation. Mean values along the row (among the hospitals) with different alphabetical superscript indicates a significant different (*p* < 0.05), while the *p*-value of less than 0.05 indicates a significant difference between the hospital services (medical and surgical);

*indicate significant difference

Table 4 shows that higher proportion (61.1%) of outpatients without a formal education had mean PE score of more than 10 points for waiting time, compared to 34.3% of outpatients with university education and 41.7% of those with high school education. The result shows significant association between PE scores on waiting time and level of education. This was not so for PE score on OPD environment, quality of doctors’ care and responsiveness of care for outpatients (as shown in Table 4). With quality of care by nurses/other care givers, the result show significant (*p* ≤ 0.05) association with educational status. Again, larger proportion of outpatient without formal education (85.7%) scored this domain high compared to 68.6% of outpatients with university education. In addition, the result shows that employment status only had significant (p ≤ 0.05) association with the overall PE rating; about 75.7% of retirees and 64.7% of employed outpatients recorded higher overall PE rating in the hospitals studied. Students and the un-employed had least overall PE rating.

**Table 4:** Measure of association between patient experience variables and socioeconomic status of outpatients

SES	Categories	Waiting Time		Chi-Square	Linear by Linear Association
		≤10	> 10		
Level of Education	No Formal Education	21^a^(38.9)	33^a^(61.1)		11.215[0.001]*
	Primary Education	47 ^b^(55.3)	38^b^(44.7)		
	Secondary Education	60^c^(58.3)	43^c^(41.7)		
	Tertiary Education	109^d^(65.7)	57 ^d^(34.3)		
Employment Status	Employed	102^a^(60)	68^a^(40)	6.358[0.95]	
	Not Employed	56 ^c^(48.7)	59^c^(51.3)		
	Student	31^a^(63.3)	18^a^(36.7)		
	Retired	48 ^b^(64.9)	26^b^(35.1)		
Variable	Categories	OPD Environment		Chi-Square	Linear by Linear Association
		≤15	> 15		
Level of Education	No Formal Education	1 ^a^(1.9)	53^a^(98.1)		2.337[0.126]
	Primary Education	4^a^(4.7)	81 ^a^(95.3)		
	Secondary Education	5^a^(4.9)	98^a^(95.1)		
	Tertiary Education	12^a^(7.2)	154^a^(92.8)		
Employment Status	Employed	14^a^(8.2)	156^a^(91.8)	4.734[0.192]	
	Not Employed	4^a^(3.5)	111^a^(96.5)		
	Student	2^a^(4.1)	47^a^(95.9)		
	Retired	2^a^(2.7)	72^a^(97.3)		
Variable	Categories	Quality of Doctors Services		Chi-Square	Linear by Linear Association
		≤25	> 25		
Level of Education	No Formal Education	5^a^(9.3)	49^a^(90.7)		0.246[0.62]
	Primary Education	8^a^(9.4)	77^a^(90.6)		
	Secondary Education	6^a^(5.8)	97^a^(94.2)		
	Tertiary Education	13^a^(7.8)	153^a^(92.2)		
Employment Status	Employed	13^b^ (7.6)	157^b^(92.4)	6.706[0.082]	
	Not Employed	12^c^(10.4)	103^c^(89.6)		
	Student	6^c^(12.2)	43^c^(87.8)		
	Retired	1^a^(1.4)	73^a^(98.6)		
Variable	Categories	Quality of care or Services by Nurses/other Medical Staff		Chi-Square	Linear by Linear Association
		≤25	> 25		
Level of Education	No Formal Education	3^a^(14.3)	18^c^(85.7)		7.132[0.008]*
	Primary Education	5^b^(20.8)	19^b^(79.2)		
	Secondary Education	10^b^(19.6)	41^b^(80.4)		
	Tertiary Education	26^c^(38.2)	41^a^(60.3)		
Employment Status	Employed	13^a^(20.3)	51^a^(79.7)	5.057[0.536]	
	Not Employed	17^a^(30.4)	39^a^(68.6)		
	Student	6^a^(40)	9^a^(60)		
	Retired	9^a^(31)	20^a^(69)		
SES	Categories	Responsiveness		Chi-Square	Linear by Linear Association
		≤50	> 50		
Level of Education	No Formal Education	18^a^(33.3)	36^a^(66.7)		0.569[0.451]
	Primary Education	33^a^(38.8)	52^a^(61.2)		
	Secondary Education	37^a^(35.9)	66^a^(64.1)		
	Tertiary Education	52^a^(31.3)	114^a^(68.7)		
Employment Status	Employed	55^a^(32.4)	115^a^(67.6)	0.675[0.879]	
	Not Employed	42^a^(36.5)	73^a^(63.5)		
	Student	18^a^(36.7)	31^a^(63.3)		
	Retired	25^a^(33.8)	49^a^(66.2)		
Variable	Categories	Overall Rating (%)		Chi-Square	Linear by Linear Association
		≤70	> 70		
Level of Education	No Formal Education	17^a^(31.5)	37^a^(68.5)		1.312[0.252]
	Primary Education	32^a^(37.6)	53^a^(62.4)		
	Secondary Education	33^a^(32)	70^a^(68)		
	Tertiary Education	68^a^(41)	98^a^(59)		
Employment Status	Employed	60^b^(35.3)	110^b^(64.7)	8.711[0.033]*	
	Not Employed	51^c^(44.3)	64^c^(55.7)		
	Student	21^c^(42.9)	28^c^(57.1)		
	Retired	18^a^(24.3)	56^a^(75.7)		

Each superscript letter denotes a subset of Variable categories whose row proportions do not differ significantly from each other at the .05 level, ()-%, [] - *p*-value

*indicate significant difference

## Discussion

The present study evaluated how outpatients of two different socioeconomic groups (educational and employment status) rate their experience in respect to various domains of care at Teaching hospitals in Southeast Nigeria. The study is an attempt to know how patient experience can serve as metric for health industry competition and differentiation, since it reflects quality of care from the patient’s perspective [21]. The study confirms that measurement of patient experience is an important aspect of evaluation of healthcare services; and it provides an opportunity to improve care, meet patients’ expectations, enhance strategic decision making, as well as effectively manage and monitor health care performance [21]. These are vital because there is no doubt that in the public eye, hospitals will continue to be the ‘face’ of the health system, and upon it the public assesses the quality of services provided [22]. Moreover, there is this emerging consensus that patient experience is a fundamental aspect of provider quality [23].

An inference from the findings is that UNTH had the shortest waiting time because patients spent less time waiting for services from doctors or other care givers. The other hospitals could learn from what UNTH is doing right and apply the same procedures in their facilities in order to decrease their waiting times and enhance PE for quality of ambulatory care. It has been shown that increased waiting time also affect the overall treatment provided by physicians and other care givers [24]. In UK the national standard or accepted waiting time is 30 min [25]. In Nigeria, previous studies show that the majority of outpatients wait as long as 80–180 min or more in the OPDs, and the commonest reason for this long waiting time is the large number of patients as against few health workers [17, 26]. A study in India obtained average waiting time outside the various OPDs of only 12 min and 98.52% of patients were very satisfied with it [13]. The finding that very educated patients, unlike the illiterate ones, are less satisfied with waiting time, could be a reflection of their increased awareness of their rights and privileges because of their level of education and therefore more discerning of the expected quality of healthcare services. The educational status of the respondents was also related to their expectations of the quality of care by the nurses/other health workers. The poorly educated outpatients rated waiting time and quality of care by nurses/other health workers higher than the more educated people, probably because they were not aware of their right to quality services and care. On the other hand, educational status was not significantly associated with patients’ rating for OPD environment, doctors’ care and responsiveness of care. However, this differs from the findings of Zalmanovitch and Vashdi (2014) who reported that a lower level of education predicates greater responsiveness of care for primary and preventive healthcare than higher level of educational status [27].

The mean overall patient experience rating in this study, though higher than that of similar study in a Federal Medical Centre in Southeast Nigeria (where average satisfaction score of outpatients respondents was 66.8%), is slightly below 83.0% obtained in a hospital of similar status (Aminu Kano teaching hospital) in Northern Nigeria [28–30]. The present study showed that employment status, and not level of education, had direct significant association with patients’ rating for overall quality of care. Higher proportion of employed and retired outpatients rated the quality of care from the hospitals high. This, to some extent, is at variance with the findings of Arpey, et al. (2017) who in their recent study observed that patients’ SES had no impact in the way they are viewed or treated by their physicians [31]. However, our findings agreed with that of Myers, et al. (2006) and Bernheim, et al. (2008) who found that physicians as a group perceive and treat low SES patients differently from those of higher SES [32, 33]. Maharlouei, et al. (2017) had also reported on how the overall patient experience was associated with different SES [34].

The limitations of this study include its inability to capture all the domains of patient care in the hospitals. It was restricted to measures of outpatient or ambulatory healthcare services. Thus, the findings cannot be generalized to include that of admitted patients. Another limitation is the fact that the data collection instrument used both coding and tick-box approaches, which could have led to some confusion for the enumerators/interviewers. However, the thorough training that was undertaken by the interviewers before field work ensured that they understood the response formats and could navigate between coding and tick-box approaches. Coding framework from previous studies was adapted [18–20] and pretested in the study area, hence the possible occurrence of bias is potentially insignificant. Although, the pre-test showed that the instrument worked well within the study context, it is recommended that a uniform coding approach be used in future studies.

## Conclusion

The study revealed how employment status was significantly associated with overall patient experience rating of the quality of care for ambulatory (outpatients) healthcare services in Nigeria hospitals. It also showed that level of education significantly impacted on how outpatients perceived waiting time and quality of care from nurses and other care givers in the hospital. Further study will be required to assess other drivers of the rating of quality of care, the provider-side influences on quality of care and the effect of socio-economic status on inpatients type of healthcare services in the hospitals.

### Recommendations

The study has underscored the need to put into consideration the socioeconomic status of patients in the course of providing healthcare services for a given population.

## Supplementary information

**Additional file 1.** Patient Experience Questionnaires (PEQ) on Quality of Healthcare Services in Nigeria Teaching Hospitals.

**Additional file 2.** Informed Consent Form for patients.

**Additional file 3.** Samples of Ethical Approval for the Study.

## Data Availability

Data-sets used and analyzed for the study are available and may be released by the corresponding author on reasonable request.

## Abbreviations

ESUTH: Enugu State University Teaching Hospital
FETHA: Federal University Teaching Hospital
LMICs: Low- and middle-income countries
MOPD: Medical outpatient department
OPD: Outpatient department
PE: Patient experience
PEQ: Patient experience questionnaire
SES: Socio-economic status
SOPD: Surgical outpatient department
UNTH: University of Nigeria Teaching Hospital
