# Supplementary material for: Impact of socioeconomic status on patient experience on quality of care for ambulatory healthcare services in tertiary hospitals in Southeast Nigeria
Source: BMC Health Serv Res. 2020 May 26;20:473. doi: 10.1186/s12913-020-05332-0 (PMC7251830; doi:10.1186/s12913-020-05332-0)
Supplement: Supplementary file 1 — Additional file 1. Patient Experience Questionnaires (PEQ) on Quality of Healthcare Services in Nigeria Teaching Hospitals. [file 12913_2020_5332_MOESM1_ESM.docx]

# *Additional File-1*

**PATIENT EXPERIENCE QUESTIONNAIRES (PEQ) ON QUALITY OF HEALTHCARE SERVICES IN NIGERIA TEACHING HOSPITALS**

Dear respondent, we are trained interviewers from Department of Health Administration & Management, University of Nigeria. We are interested in estimating the quality of healthcare services you get from our teaching hospitals and we wish to do so through your opinion. To achieve this aim, we wish to conduct a questionnaire survey on your perception of healthcare services as provided by this particular Teaching hospital. You shall in the future get the results of this exercise, and we hope to use your responses to help you get desired healthcare services in the future. Please be confident to answer what you really feel, since whatever you answer may affect the future improvement of these services. All information given will be private. Your participation is voluntary, and you do not have to answer questions you do not feel like.

This questionnaire is to be filled by outpatients visiting Medical Outpatient Department (MOPD) and Surgical Outpatient Department (SOPD) of the Teaching Hospitals. For most questions, please tick clearly inside one box using a black or blue pen. Don’t worry if you make a mistake; simply cross out the mistake and put a tick in the correct box. Please, do not write your address anywhere on the questionnaire. **If you have questions or need help,** please call the helpline phone number written below.

**Preliminary Section: BIODATA AND PRELIMINARY QUESTIONS**

**Date of Interview: Start Time: Time End:**

**Hospital Name: …………………………….**

**Outpatient Dept.**: ………..

1. Sex: a) Write 1 for male and 0 for female; Male [ ] b) Female [ ]
2. Age (as at last birthday)…………….
3. Level of Education: Write 0 or 1 or 2 or 3 accordingly; No formal education [ ], Primary Education [ ], Secondary Education [ ], University Education [ ].
4. Marital Status: Write 3, 2, 1, 0 accordingly; Married [ ], Not Married [ ], Divorced [ ], Widow [ ].
5. Employment status: Write 3, 2, 1, 0 accordingly; Employed [ ]; Not Employed [ ]; Student [ ]; Retired [ ].
6. Religion: Write 1 for Christian and 0 for Muslim, Christian [ ]; Muslim [ ].
7. Are you living in the same State where the hospital you are attending is located?

Write 1 for ‘yes’ and 0 for ‘no’; Yes [ ], No [ ].

1. Have you ever visited the Outpatients Department of this Teaching Hospital before and for the same condition or any other condition? Write 1 for ‘yes’ and 0 for ‘no’. Yes [ ]; No [ ].

**Section (a) ARRIVAL/WAITING AT OPD AND HOSPITAL ENVIRONMENT (60 points)**

**1. How long did it take you to get from home to Outpatients Department of the hospital?**

1.1 Less than 30 minutes 10

1.2 31 to 60 minutes 3

1.3 More than 1 hour but not more than 2 hours 2

1.4 More than 2 hours 0

1.5     Don’t know/can’t remember Not applicable

**2. How long after you arrived did it takes you to retrieve or obtain folder.**

2.1 Was attended to immediately 10

2.2 Waited up to 10 minutes 8

2.3  Waited up to 30 minutes 5

2.4  Waited up to 60 minutes 2

2.5. Waited more than an hour (60minutes) 0

2.6  Don’t know/can’t remember Not applicable

**3. How long did you wait before seeing the doctor?**

3.1 The waiting was short, less than 20minutes 10

3.2 I had to wait as long as 30 – 60minutes 5

3.3 Waited for as long as 1 – 2 hours 3

3.4 I waited more than 2 hours before seeing a doctor 2

3.5 Don’t know/can’t remember Not applicable

**4. Were you able to find a place to sit in the waiting area?**

4.1 Yes, I found a place to sit straight away 10

4.2  Yes, but I had to wait for a seat 5

4.3  No, I could not find a place to sit 0

4.4 I did not want to find a place to sit Not applicable

4.5    Don’t know/can’t remember Not applicable

**5. Was the waiting area in the open or inside a building?**

5.1 Yes, it was inside 10

5.2  No, it was in the open but with a shelter 5

5.3  No, it was in open space without a shelter 0

5.4 Don’t know/can’t remember Not applicable

**6. In your opinion, how clean was the Outpatients Department?**

6.1  Very clean 10

6.2 Fairly clean 5

6.3  Not clean 0

6.4 Can’t say Not applicable

**Section (b) SEEING A DOCTOR (50 points)**

**7. How long were you with the doctor?**

7.1 Less than 10minutes 3

7.2 11 - 20 minutes 5

7.3  21- 30 minutes 10

7.4 31- 40 minutes 5

7.5 More than 40 minutes 2

7.6 Can’t remember Not applicable

**8. Did you have enough time to discuss your health or medical problem with the doctor?**

8.1  Yes, definitely 10

8.2 Yes, to some extent 5

8.3    No 0

**9. Did the doctor listen to what you had to say?**

9.1  Yes, definitely 10

9.2 Yes, to some extent 5

9.3  No 0

**10. Did the doctor explain the reasons for any treatment or action in a way that you could understand?**

10.1 Yes, completely 10

10.2 Yes, to some extent 5

10.3 No 0

10.4 I did not need an explanation Not applicable

10.5 No treatment or action was needed Not applicable

**11. Did you have confidence and trust in the doctor examining and treating you?**

11.1 Yes, definitely 10

11.2 Yes, to some extent 5

11.3 No 0

**Section (c) SEEING OTHER MEDICAL PROFESSIONALs (30 points)**

- **Was all or part of your Outpatient appointment with any member of staff, other than a doctor?**

a. Yes 0

b. No 0

If ‘No’ skip12, 13 and 14.

- **Who was the MAIN person, other than a doctor, you saw? (Tick ONE ONLY)**

a. A nurse 0

b. A Laboratory scientist 0

c. A radiographer 0

d. Someone else (**Please write in box)**

**12. How long were you with him/her?**

12.1  Up to 5 minutes 5

12.2 6-10 minutes 10

12.3  11-20 minutes 7

12.4  21-30 minutes 3

12.5  More than 30 minutes 0

12.6  Can’t remember Not applicable

**13. Did he/she explain the reasons for any treatment or action in a way that you could understand?**

13.1  Yes, definitely 10

13.2 Yes, to some extent 5

13.3  No 0

13.4 I did not want an explanation Not applicable

13.5  No treatment or action was needed Not applicable

**14. Did you have confidence and trust in him/her?**

14.1  Yes, definitely 10

14.2 Yes, to some extent 5

14.3  No 0

**Section (d): CLINICAL INVESTIGATIONS (e.g. Lab. & Radiological, etc) 30 points**

**15. Did you have any tests (such as x-rays, ultrasound scans or blood/urine, etc tests)?**

15.1 Yes 10

15.2 No 0

If No, skip 16 and 17 below.

**16. Did a member of staff explain why you needed these test (s) in a way you could understand; and did he/she tell you how and when you would find out the results of your test(s)?**

16.1 Yes, completely 10

16.2 Yes, to some extent 5

16.3 No 0

16.3 Not sure/can’t remember Not applicable

16.4 I did not need an explanation Not applicable

**17. Did a member of staff explain the results of the tests in a way you could understand?**

17.1 Yes, definitely 10

17.2 Yes, to some extent 5

17.3   No 0

17.4 I was never told the results of the tests 0

17.5 Not sure/can’t remember 0 Not applicable

**Section (e) TREATMENT (20 points)**

*By treatment we mean any medical or surgical intervention, procedure or drug prescription (therapy).*

- **During your Outpatient appointment, did you have any treatment for your condition?**

a. Yes 0

b. No 0

If NO, skip 18 and 19.

**18. During prescription by doctor, were you involved as much as you wanted to be in decisions about the best medicine for you?**

18.1 Yes, definitely 10

18.2 Yes, to some extent 5

18.3. No 0

**19. Before the treatment did a member of staff explain the dosage, the risks/side effects and the benefits of the treatment in a way you could understand?**

19.1 Yes, definitely 10

19.2 Yes, to some extent 5

19.3 No 0

19.4 I did not want explanation Not applicable

**Section (f): OVERALL ASSESMENT OF YOUR VISIT TO OUTPATIENNT DEPARTMENT (70points)**

**20. Did doctors and/or other staff talk in front of you as if you weren’t there?**

20.1 Yes, definitely 10

20.2 Yes, to some extent 5

20.3 No 0

**21. Sometimes in a hospital or clinic, a member of staff will say one thing and another will say something quite different. Did this happen to you?**

21.1 Yes, definitely 0

21.2 Yes, to some extent 5

21.3 No 10

**22. How much information about your condition or treatment was given to you?**

22.1 Not enough 0

22.2 Right amount 10

22.3 Too much 5

22.4 I was not given any information about my treatment or condition 0

**23. Were you involved as much as you wanted to be in decisions about your care and treatment?**

23.1 Yes, definitely 10

23.2 Yes, to some extent 5

23.3 No 0

**24. Were you given enough privacy when discussing your condition or when you were being examined or treated?**

24.1 Yes, definitely 10

24.2 Yes, to some extent 5

24.3 No 0

**25. Were you asked for permission for medical students to be present when you were being treated or examined?**

25.1 Yes 10

25.2 No 0

25.3 This was not necessary Not applicable

**26. Did doctors and/or staff tell you what was important to you in managing your condition or illness?**

26.1 Yes, definitely 10

26.2 Yes, to some extent 5

26.3 No, but I would have liked this 0

26.4 This was not necessary Not applicable

**Section (g): OVERALL IMPRESSION (40points)`**

**27. Was the main reason you went to the Outpatients Department dealt with to your satisfaction?**

27.1 Yes, completely 10

27.2 Yes, to some extent 5

27.3 No 0

**28. How well organized was the Outpatient Department you visited?**

28.1 Not at all organized 0

28.2 Fairly organized 5

28.3 Very well organized 10

**29. Overall, were you treated with understanding, respect and dignity, while you were at the Outpatients Department?**

29.1 Yes, all of the time 10

29.2 Yes, some of the time 5

29.3 No 0

**30. Overall, how would you rate the services or care you received at the Outpatients Department?**

30.1  Excellent 10

30.2 Very good 8

30.3 Good 5

30.4  Fair 3

30.5 Poor 1

30.6 Very poor 0

**Section h. ANY OTHER COMMENTS**

**31. If there is anything else you would like to tell us about your experiences in the Outpatients Department, please do so here.**

Was there anything particularly good about your visit to the Outpatients Department?

Was there anything that could have been improved?

Any other comment?

***For further clarification contact us on: 08033097760 AND*** [***henryealoh@gmail.com***](mailto:henryealoh@gmail.com)

# *Appendix-2*

## Table 2: Reliability Test for Patient Questionnaire using Cronbach Alpha

| Items | Item statistics | | | Item total Statistics | | | | | |
| --- | --- | --- | --- | --- | --- | --- | --- | --- | --- |
|  | Mean | Std. Deviation | | Scale Mean if item deleted | Scale Variance if item deleted | Corrected Item-Total Correlation | | Squared Multiple Correlation | Cronbach's Alpha |
| Sex | 1.53 | .500 | | 489.60 | 5689.436 | 0.024 | | 0.111 | 0.635 |
| Age | 47.55 | 18.118 | | 443.58 | 5033.447 | 0.128 | | 0.358 | 0.647 |
| Level of Education | 3.01 | 1.039 | | 488.13 | 5702.762 | -0.078 | | 0.202 | 0.636 |
| Marital Status | 1.37 | .578 | | 489.76 | 5703.405 | -0.140 | | 0.194 | 0.636 |
| Employment Status | 2.06 | 1.096 | | 489.08 | 5697.779 | -0.045 | | 0.235 | 0.636 |
| Residence | 1.29 | .454 | | 489.84 | 5689.537 | 0.026 | | 0.226 | 0.635 |
| Number of Visit | 1.73 | .445 | | 489.40 | 5677.838 | 0.201 | | 0.225 | 0.634 |
| Travelling Time | 4.67 | 3.715 | | 486.46 | 5714.447 | -0.065 | | 0.216 | 0.639 |
| Waiting Time | 9.89 | 5.213 | | 481.24 | 5575.272 | 0.114 | | 0.211 | 0.631 |
| OPD Environment | 26.28 | 4.601 | | 464.85 | 5392.140 | 0.412 | | 0.272 | 0.615 |
| Quality of Doctors services | 41.71 | 7.943 | | 449.43 | 5032.772 | 0.529 | | 0.456 | 0.591 |
| Quality of Services by Other Medical Staff | 21.07 | 7.245 | | 470.06 | 5276.230 | 0.345 | | 0.397 | 0.611 |
| Responsiveness | 46.72 | 14.179 | | 444.42 | 4307.743 | 0.635 | | 0.665 | 0.543 |
| Overall | 209.28 | 38.635 | | 281.86 | 1585.241 | 0.850 | | 0.918 | 0.504 |
| Rating | 72.98 | 11.530 | | 418.15 | 4247.674 | 0.872 | | 0.863 | 0.518 |
|  | | | |  | | | | | |
| ***Reliability Statistic*** | | | | | | | | | |
|  | Cronbach's Alpha | | Cronbach's Alpha Based on Standardized Items | | | | Number of Items | | |
|  | 0.632 | | 0.518 | | | | 15 | | |
