# Supplementary material for: Impact of socioeconomic status on patient experience on quality of care for ambulatory healthcare services in tertiary hospitals in Southeast Nigeria
Source: BMC Health Serv Res. 2020 May 26;20:473. doi: 10.1186/s12913-020-05332-0 (PMC7251830; doi:10.1186/s12913-020-05332-0)
Supplement: Supplementary file 2 — Additional file 2. Informed Consent Form for patients. [file 12913_2020_5332_MOESM2_ESM.docx]

**DEPARTMENT OF HEALTH MANAGEMENT AND ADMINISTRATION**

**FACULTY OF HEALTH SCIENCES**

**UNIVERSITY OF NIGERIA**

**ENUGU CAMPUS**

**INFORMED CONSENT FORM FOR OUTPATIENTS**

IRB Research Approval No. .........................................

This Approval will elapse on: ......................................

***TITLE OF RESEARCH:***

Evaluating the Quality of Healthcare Services, Levels and Determinants of Efficiency in Nigeria Teaching Hospitals: A Mixed Methods Study

***NAME AND AFFIALIATION OF RESEACHER:***

This study is being conducted by Dr. Henry E. Aloh of the Federal University Ndufu-Alike Ikwo, under the supervision of Prof. Obinna E. Onwujekwe of Department of Health Administration and Management, University of Nigeria, Enugu Campus.

***SPONSOR:*** Self

***PURPOSE OF RESEARCH:*** The purpose of this research is to evaluate the quality of healthcare services and the level of efficiency among Nigeria University Teaching hospitals, so as to understand critical issues for improving performance in such hospitals.

***PROCEDURE OF THE RESEARCH, WHAT SHALL BE REQUIRED OF EACH PARTICIPANT AND APPROXIMATE TOTAL NUMBER OF PARTICIPANTS THAT WOULD BE INVOLVED IN THE RESEARCH:*** As a participant you are only required to fill the outpatient’s experience questionnaire on your own or with the assistance of a trained interviewer. About a total of 200 medical and surgical outpatients are expected to participate in this study. As a participant you are to confidently answer what you really feel, since whatever you answer may affect the future improvement of healthcare services in this teaching hospital and in Nigeria at large. All information given will be private. Your participation is voluntary, and you do not have to answer questions you do not feel like. The questionnaire is to be filled by you as an outpatient visiting Medical Outpatient Department (MOPD) or Surgical Outpatient Department (SOPD) of this Teaching Hospital. For most questions, please tick clearly inside one box using a black or blue pen. Don’t worry if you make a mistake; simply cross out the mistake and put a tick in the correct box. Please, do not write your address anywhere on the questionnaire. **If you have questions or need help,** please call the helpline phone number written at the end of the questionnaire.

***EXPECTED DURATION OF RESEARCH AND OF PARTICIPANTS INVOLVEMENT:***

You are to be interviewed at the end of this visit in the outpatient department. The interview will last between 15mins and 1 hour, except when interrupted.

***RISKS:*** No risk is anticipated.

***COSTS TO THE PARTICIPANTS:*** The only cost for is the short time you will spend for the interview.

***BENEFIT(S):*** The purpose of the research is to know the actual performance of Nigeria teaching hospitals and to determine in what ways to improve this.

***CONFIDENTIALITY:*** All information collected in this study will be given code numbers and no name will be recorded. It cannot be linked to you in anyway and your name or any identifier will not be used in the reports or any publications. However, as part of our responsibility to conduct this study properly, official of Federal Ministry of Health, especially their ethics committee may have access to these records.

***VOLUNTARINESS:*** Your participation in this research is voluntary.

***ALTERNATIVE TO PARTICIPATION:*** If you choose not to participate, this will not affect your treatment or care in this hospital.

***CONFLICT OF INTEREST:*** The researcher does not work in any of the Teaching Hospitals and there is no conflict of interest.

***STATETMENT OF PERSON (INTERVIEWER) OBTAINING INFORMED CONSENT:***

I have fully explained this research to ........................................................................................... and have given sufficient information, including about risks and benefits, to make an informed decision.

DATE: ..................................... SIGNATURE: ...................................................................................................

NAME: ............................................................................................................................................................

***STATEMENT OF PERSON GIVING CONSENT:***

I have read the description of the research or have had it translated into language I understand. I have also talked it over with the interviewer to my satisfaction. I understand that my participation is voluntary. I know enough about the purpose, methods, risks and benefits of the research study to judge that I want to take part in it. I understand that I may freely stop being part of this study at any time. I have received a copy of this consent form to keep for myself.

DATE: ...................................... SIGNATURE: ..............................................................

NAME: ........................................................................................................................

**DETAILED CONTACT INFORMATION OF I-HREC, RESEARCHER AND H.O.D. OF THE DEPARTMENT:**

1. Prof. O. U. J. Umeora

Federal Teaching Hospital Abakaliki,

[oujair@yahoo.com](mailto:oujair@yahoo.com) ; +2348039558074 or +27834049650

1. Dr. Henry E. Aloh

FUNAI Medical Centre, Federal University Ndufu-Alike Ikwo

Ebonyi State

[henryealoh@gmail.com](mailto:henryealoh@gmail.com); 08033097760

**3)** Dr. (Mrs.) Ijeoma L. Okoronkwo

The Head of Department

Dept. of Health Administration and Management

University of Nigeria, Enugu Campus

0806 358 1297
