# Supplementary figures and images for: Impact of socioeconomic status on patient experience on quality of care for ambulatory healthcare services in tertiary hospitals in Southeast Nigeria
Source: BMC Health Serv Res. 2020 May 26;20:473. doi: 10.1186/s12913-020-05332-0 (PMC7251830; doi:10.1186/s12913-020-05332-0)

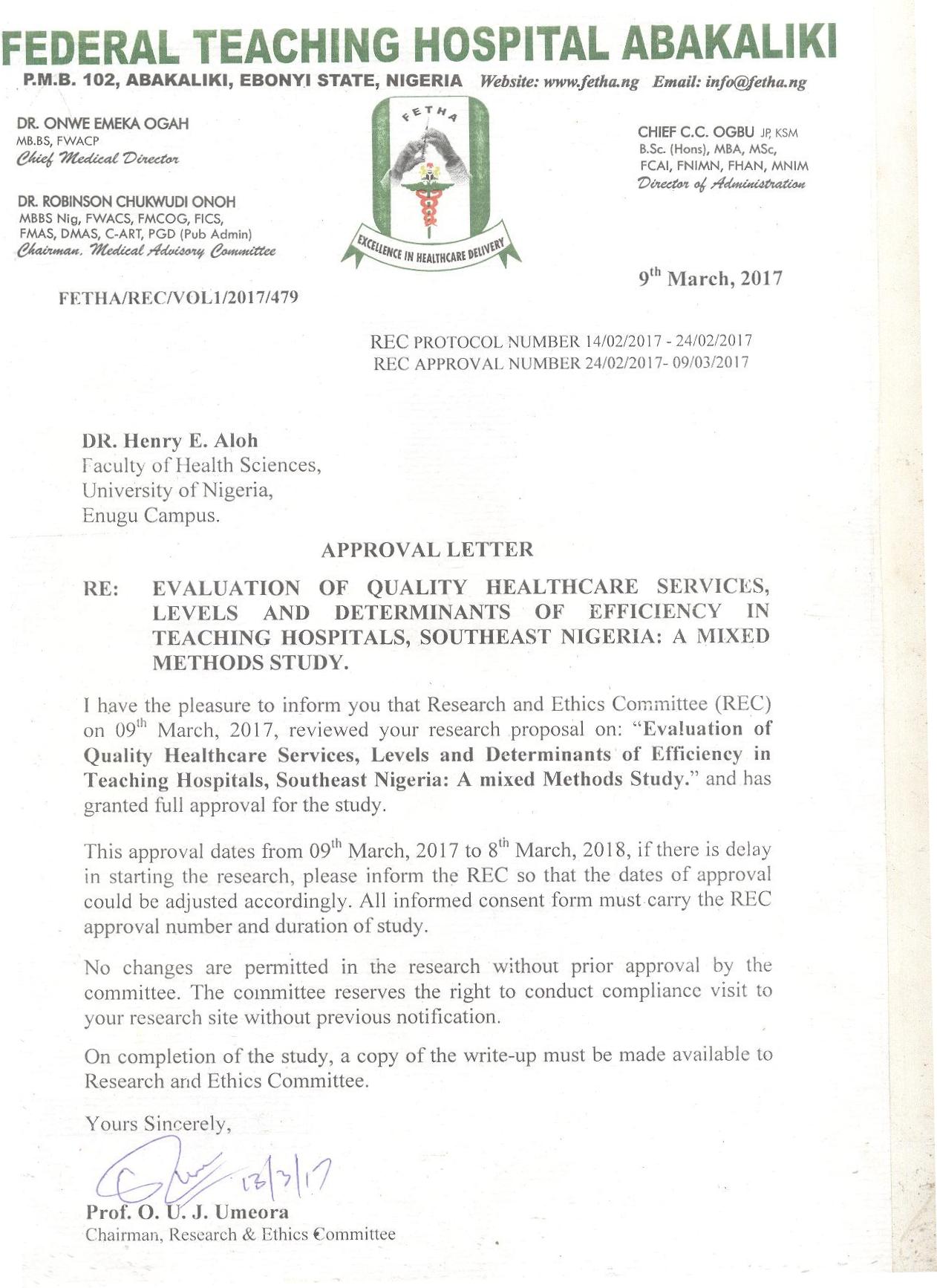


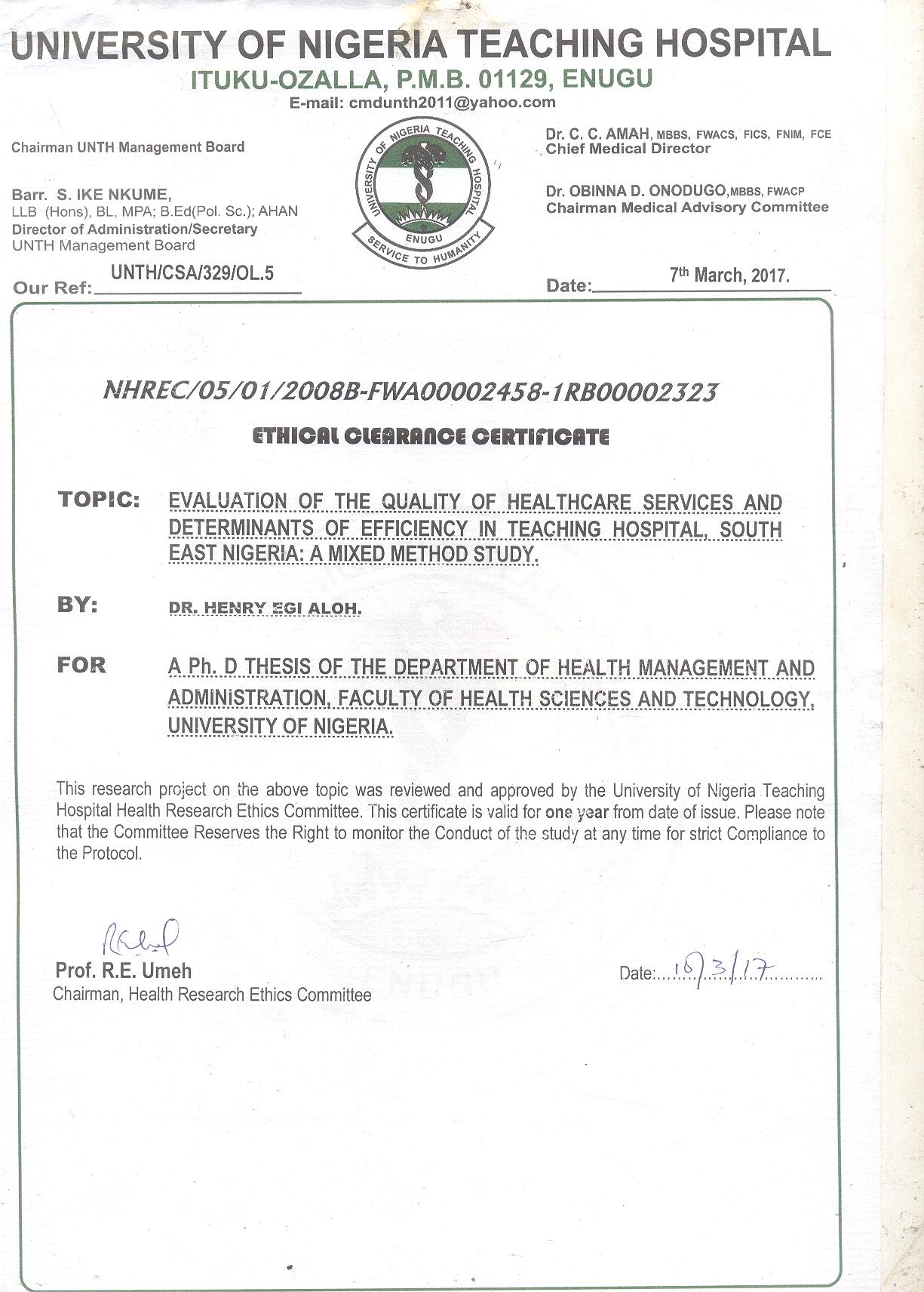

Supplement: Supplementary file 3 — Additional file 3. Samples of Ethical Approval for the Study. [file 12913_2020_5332_MOESM3_ESM.docx]
